# Is Vesicular Therapy the Newcomer That Matters for the Medicine of Tomorrow?

**DOI:** 10.3390/ijms25063530

**Published:** 2024-03-20

**Authors:** Émilie Velot, Arnaud Bianchi

**Affiliations:** IMoPA (Molecular Engineering and Articular Physiopathology), CNRS (French National Centre for Scientific Research), Université de Lorraine, F-54000 Nancy, France

Extracellular vesicles (EVs) are membrane-enclosed particles released by cells into their extracellular environment. These vesicles play a crucial role in intercellular communication, facilitating the transfer of various bioactive molecules, including proteins, lipids, and nucleic acids, between cells. As there is still no codified nomenclature, EVs can be categorized according to various features: e.g., their biogenesis (such as exosomes from the endosomal pathway) or their size (such as small or large EVs) [1]. The cargo carried by EVs reflects the biological state of the parent cell, influencing recipient cells in various physiological and pathological processes [2]. Research on EVs has expanded rapidly these last twenty years due to their potential diagnostic and therapeutic applications [3]. EVs participate in many cellular functions, such as immunomodulation, tissue repair, and the progression of various diseases, including cancer [4]. The emerging field of vesicular therapy holds promise for advancing our knowledge of intercellular communication and developing innovative therapeutic strategies, such as personalized drug delivery systems [5,6].

The aim of this Special Issue was to shine a light on research that examines these developments with nine contributions (four research articles and five reviews) mentioning EVs in both diagnostic and therapeutic approaches through various systems (cardio/neurovascular, respiratory, and musculoskeletal), cancer, and regenerative medicine.

This editorial provides a concise overview of these research articles and reviews, summarizing their key findings and implications.

EVs have been recognized as potential biomarkers for diagnosing diseases, including cardiac conditions [7]. In the context of the heart, different cell types, including cardiomyocytes, fibroblasts, and endothelial cells, communicate through EVs to maintain cardiac homeostasis. Contribution 1 emphasized that the number, size, and content of cardiac EVs can vary under different pathological conditions, influencing cardiac remodeling processes such as hypertrophy, fibrosis, and inflammation. Their study investigates EVs as transporters of biomarkers for diagnosing cardiac diseases to address the lack of specific markers for the isolation and analysis of cardiac EVs. The aim of their work was to detect specific protein markers that can distinguish cardiac EVs from non-cardiac EVs in the circulation and differentiate EVs derived from various cardiac cell types. Lim domain binding 3 (Ldb3), a cytoskeletal protein essential for maintaining Z-disc structural integrity, was identified as enriched in both large and small cardiac EVs compared to plasma-derived EVs. Ldb3 was almost exclusively detected in the neonate rat heart compared to other tissues and specifically in cardiomyocytes compared to cardiac fibroblasts. Despite the challenges of EV characterization due to their heterogeneity and the lack of fully specific isolation methods, contribution 1 highlighted the importance of understanding the role of EVs in both physiological and pathological conditions.in the heart and the potential utility of Ldb3 in this context.

Trauma frequently causes severe hemorrhage leading to trauma-induced coagulopathy (TIC) [8]. TIC participates in organ failure and increases mortality. Recent research emphasizes the role of the endothelium in TIC with endothelial glycocalyx degradation, endothelial cell activation after traumatic injury and hemorrhagic shock. Contribution 2 focused on EVs isolated from plasma. They hypothesized that shock-driven endotheliopathy following trauma. is linked to high EV concentrations and exacerbated by red blood cell (RBC) transfusion. Their study showed that patients experiencing shock exhibited increased levels of markers essential for endothelial cell activation. Additionally, patients with shock had elevated concentrations of leucocyte-derived EVs and RBC-derived EVs compared to patients without shock. Although RBC transfusion increased the circulation of RBC-derived EVs, it did not affect endotheliopathy, indicating that RBC transfusion may not exacerbate endothelial activation in this context. Endotheliopathy markers were associated with leucocyte-derived EVs, suggesting glycocalyx degradation by an immune-driven pathway in trauma patients. These findings propose that shock is a primary driver of endotheliopathy after trauma associated with increased leucocyte derived EVs and that the modulation of leucocyte immune response post-trauma may have a protective effect on the endothelial glycocalyx and may impact patient outcomes.

Ischemic stroke results from a restricted blood supply, leading to oxygen and nutrient shortages, often due to blood clot formation or emboli from other body parts. This obstruction causes energy failure in cells, leading to intracellular accumulation of ions and mitochondrial dysfunction. This cascade can trigger cell death in the ischemic core, while surrounding areas may survive with timely reperfusion [9]. Despite experimental treatment targets, clinical success remains limited, prompting exploration of novel approaches. Ischemic conditioning and exercise-induced ischemic tolerance, such as blood-flow-restricted resistance exercise (BFRRE) or high-load resistance exercise (HLRE), show promise in promoting ischemic tolerance. These methods may share protective mechanisms with remote ischemic conditioning (RIC). EVs, including exosomes, are implicated in intercellular communication and have been proposed as mediators of protective effects. Muscle-specific EVs, increased during exercise and may contribute to neuroprotection. Contribution 3 explored the capacity of EVs released in plasma to mediate protection against brain ischemia-reperfusion injury. EVs were isolated from individuals undergoing RIC, BFRRE, and HLRE and tested on endothelial cells. Post-RIC and post-BFRRE EVs improved the viability of endothelial cells subjected to oxygen-glucose deprivation, suggesting a potential protective role. Despite in vitro protection, the results showed that post-RIC EVs did not significantly preserve blood-brain barrier (BBB) integrity in a mouse model of ischemic stroke, possibly due to early-phase BBB breakdown mechanisms. While conditioned EVs did not significantly alter tube formation in angiogenesis assays, in vivo studies show post-RIC EVs localize to ischemic brain areas, indicating a potential homing effect. The study indicated potential acute tissue protective effects of RIC and BFRRE, influencing EV composition and function. Whereas post-RIC EVs exhibited a protective effect in vitro, they failed to demonstrate significant neuroprotection or functional improvement in acute ischemic stroke. Contribution 3 suggested that circulating EVs induced by RIC and BFRRE can mediate protection, but further investigations are needed to understand the complex interplay between EVs and ischemic tolerance, potentially unlocking neuroprotective molecules for direct administration and for EV use to target drug delivery to ischemic brain areas.

Osteoarthritis (OA) is a degenerative disease associated with articular pain and reduced functional capacities, primarily attributed to the degradation of cartilage matrix and downregulation of lubricating molecules. Current treatments, including autologous chondrocyte transplantation (ACT), face challenges related to chondrocyte dedifferentiation and limited curative outcomes [10]. Contribution 4 investigated the use of bone marrow MSCs, specifically their secretome, which includes soluble mediators and EVs, to potentially enhance chondrocyte repair and mitigate inflammation. The authors noted that while the differentiation of MSCs into chondrocytes is an attractive approach, their incomplete differentiation status remains a limitation. The secretome, comprising factors released by MSCs, demonstrates potential in promoting tissue repair and immunomodulation. The potential therapeutic impact of the secretome was evaluated on equine articular chondrocytes (eACs) in the context of equine OA. The investigation involved co-culture experiments of MSCs and eACs, exploring both direct and indirect interactions. It also assessed the impact of MSC-conditioned media (MSC-CM) on cartilage organoids, chondrocyte migration, and the potential presence of EVs, particularly exosomes, in MSC-CM. The results showed that MSC secretome, including MSC-CM, influences eACs by increasing mRNA levels cartilage functionality markers, and proliferation-associated molecules. Furthermore, MSC-CM did not exhibit cytotoxic effects and enhanced eAC matrix synthesis. Contribution 4 suggested that MSC-CM promotes chondrocyte migration, potentially facilitating tissue repair. Although the authors emphasized the potential of MSC secretome for developing innovative therapeutic strategies for equine OA, they acknowledged the need for further investigations, particularly in optimizing the use of MSC-CM and understanding the specific molecules or EVs responsible for the observed effects.

In the rapidly evolving landscape of EV research, a cluster of five impactful reviews has been published in this Special Issue, shedding light on diverse aspects of EV biology and potential applications in cardiovascular disease (CVD; contribution 5), respiratory disorders (contribution 6), cancer diagnosis (contribution 7), and clinical therapeutics (contributions 8 and 9). The key points of these reviews are mentioned hereafter.

Mitochondrial health is critical for cardiac cellular homeostasis and a part of their content can be found in EVs. Contribution 5 examined the intricate connections between mitochondrial-derived vesicles (MDVs) and EVs, offering valuable insights into their roles in CVD. By investigating the formation, trafficking, and potential therapeutic applications of MDVs, the authors also provided a comprehensive understanding of how these vesicles may contribute to mitochondrial dysfunction in CVD. Moreover, the discussion on vesicle-mediated mitochondrial transfer introduces a novel perspective on cardioprotective strategies.

Contribution 6 explored the role of EVs as both contributors to and potential biomarkers for respiratory disorders, specifically cystic fibrosis (CF) and chronic obstructive pulmonary disease (COPD). By unraveling the intricate interplay of EVs secreted by resident or attached cells in the airways during the evolution of pulmonary pathophysiology, the authors highlighted the potential of EVs as diagnostic biomarkers for CF and COPD and offered a hint into their potential applications in respiratory medicine.

Addressing a critical aspect of cancer research, Contribution 7 emphasized advanced detection strategies for tumor-derived exosomes. By analyzing the relationship between exosomes and tumor progression, the authors provided an exhaustive summary of innovative detection methods from non-invasive liquid biopsy. The accent on single exosome phenotyping, a technique showing promise in tumor typing through serum exosome analysis, underscored the potential for early cancer diagnosis and personalized treatment.

Focusing on the clinical applications of EVs, Contribution 8 delved into critical aspects such as cell sources, isolation methods, storage, and delivery strategies. Acknowledging the impact of the coronavirus disease of 2019 (COVID-19) pandemic on EV-based therapies, the authors presented a comprehensive overview of the current state of EV therapies in clinical trials. By addressing challenges and highlighting the therapeutic potential of EVs in various diseases, the authors wrote a valuable resource for researchers and clinicians navigating the therapeutic landscape of EVs associated with COVID-19.

Contribution 9 also focused on the clinical applications of EVs by providing a thorough examination of EVs derived from MSCs in the context of regenerative medicine. The authors offered a clear outline of the therapeutic potential of MSC-derived EVs, emphasizing their regenerative effects and molecular mechanisms. The discussion on the obstacles and considerations in utilizing MSC-derived EVs added depth to our knowledge of these promising therapeutic agents.

In summary, the references included in this Special Issue collectively contribute to the growing body of knowledge surrounding EVs, spanning their roles in disease pathogenesis, therapeutic applications, and diagnostic potential. As researchers continue to unravel the complexities of EV biology, these research articles and these reviews serve as valuable resources guiding future investigations and applications in the dynamic field of vesicular strategies in health and diseases.
